# Polymorphism of MTHFR C677T Gene and the Associations with the Severity of Essential Hypertension in Northern Chinese Population

**DOI:** 10.1155/2020/1878917

**Published:** 2020-10-14

**Authors:** Junli Song, Jie Hou, Qiang Zhao, Xuezhi Liu, Qian Guo, Donghong Yin, Yan Song, Xiaoxia Li, Shuyun Wang, Xinchun Wang, Jinju Duan

**Affiliations:** ^1^Department of Pharmacy, Second Hospital of Shanxi Medical University, Taiyuan, Shanxi 030001, China; ^2^Department of Pharmacy, Shanxi Provincial Cancer Hospital, Taiyuan, Shanxi 030001, China; ^3^Department of Hospital Infection Control, Linfen Central Hospital, Linfen, Shanxi 041000, China

## Abstract

**Objective:**

Methylenetetrahydrofolate reductase (MTHFR) is a key enzyme of homocysteine metabolism and is closely related to the occurrence of hypertension. The aim of this study was to investigate the polymorphism of the (MTHFR) C677T and the factors influencing the severity of hypertension. *Material and Methods*. A total of 985 subjects were enrolled to analyze the polymorphisms of the MTHFR C677T gene by polymerase chain reaction (PCR). 306 people with essential hypertension were selected from 985 subjects to estimate the severity of hypertension by the ordinal multivariate logistical regression model.

**Results:**

The frequencies of CC, CT, and TT genotypes were 19.5%, 49.95%, and 30.46%, respectively. The allelic frequency of mutant *T* was 55.43%. The plasma homocysteine level of the homozygous TT in individuals was significantly higher than in those with CC or CT (*P* < 0.01). MTHFR677CT genotype, MTHFR677TT genotype, smoking, family history of hypertension, Hcy, and triglycerides (TG) were independent risk factors for the severity of hypertension (OR = 2.29, 2.24, 2.04, 1.81, 1.04, 1.26).

**Conclusion:**

MTHFR gene, smoking, family history of hypertension, Hcy, and triglycerides could be important genetic and high-risk factors of the development of severe hypertension in northern Chinese. These factors will contribute to the identification of high-risk populations of hypertension and facilitate the development of hypertension control strategies.

## 1. Introduction

Methylene-tetrahydrofolate reductase (MTHFR) is a key enzyme involved in the homocysteine (Hcy) metabolic pathway that catalyzes the conversion of 5, 10-methylene tetrahydrofolate into 5-methyl tetrahydrofolate. MTHFR provides a methyl group for Hcy remethylation into methionine and maintains the normal levels of Hcy in the body. Mutations of the MTHFR gene decreases MTHFR enzyme activity, preventing Hcy remethylation and eventually resulting in Hcy accumulation in the body [1]. The distribution of MTHFR C677T varies amongst different geographical regions and ethnicities. It has been reported that the distribution of *T* alleles is the highest amongst the European population (24.5%–43.8%), followed by the Asian population (2.5%–36%) and African population (4.9%–9.1%) [2]. In terms of ethnic distribution, a previous study confirmed the differences in MTHFR C677T gene polymorphisms amongst 9 ethnic groups in China [3]. In the present study, the frequency of C677TT homozygous mutant genotypes in different provinces of China was investigated.

MTHFR C677T is the most common mutated site of MTHFR [4], and the mutant allele (TT) can increase the risk of high Hcy levels [1]. The Hcy concentration of the MTHFR677-TT genotype is higher than that found in the 677-CC genotype [5]. Furthermore, increased Hcy levels are associated with an increased risk of a wide range of diseases. Fluctuations in Hcy levels by 5 mol/l increases the risk of ischemic heart disease by 32% [6]. The mortality and recurrence rate in stroke patients with Hcy levels ≥16 mol/l is 1.47 times and 1.31 times higher compared with patients with normal Hcy levels, respectively [7].

The Shanxi region, which has a total area of 155,700 km^2^ and a population of 37,0235 million, is located in the northern part of China. The local diet is dominated by grains, which are rich in B vitamins and folate, and accounts for 78% of an individual's energy intake in the region [8]. B vitamins and folate, some of which are cofactors in Hcy metabolism, can significantly reduce Hcy concentration. Furthermore, a previous study reported that the detection rate of hyperhomocysteinemia in individuals >55 years old in Luliang of the Shanxi Province reached 72.4% [9], whereas the prevalence rate in Taiyuan, the capital of Shanxi Province, was as high as 77.9%. Considering that MTHFRC677T polymorphisms are an important determinant of Hcy concentration, it was hypothesized that the high incidence of hyperhomocysteinemia in Shanxi was related to polymorphisms of MTHFRC677T. The identification of genetic predispositions may contribute to the development of personalized risk prediction and health care strategic planning. Thus, the aim of the present study was to investigate the genetic polymorphisms of MTHFRC677T and its relationship with Hcy in a cohort of patients from the Shanxi province.

Currently, there are ∼270 million patients with hypertension in China, and the prevalence rate is still increasing. Hypertension with elevated Hcy levels (h-type hypertension, ≥10 *μ*mol/l) accounts for a large proportion of cases. H-type hypertension accounts for 82.8% of the hypertensive population in Shanxi, and this is higher than the general average in China, which was found to be 75% as indicated in “Expert Consensus on Diagnosis and Treatment of H–type Hypertension.” Several studies have evaluated the association between the MTHFRC677T polymorphism, Hcy, and hypertension. Nevertheless, the reported results are inconclusive, likely due to the confounding effects of lifestyle, regions, and ethnicity. A meta-analysis that included Caucasian and Asian populations showed that carriers of the 677T allele were more susceptible to essential hypertension (EH) [10]. A study that included 347 patients with EH in the Anhui province of China revealed that the MTHFR gene polymorphism only affected the baseline diastolic blood pressure (DBP) levels in patients with EH [11].

The aim of the present study was to address several important questions related to the role of the MTHFR C677T gene in hypertension and fill in critical data gaps in the field. First, whether a high incidence of hyperhomocysteinemia could be determined by individual MTHFR genetic background was determined, as the lifestyle and diet in Shanxi are beneficial to the reduction of Hcy levels. Second, the effect of the path “MTHFR C677T gene-homocysteine-hypertension” in the grading of hypertension level (hypertension severity) was explored. Finally, multiple factors that affect the severity of hypertension were assessed, thus identifying indicators that may be used to predict the levels of hypertension and provide valuable data for physicians to evaluate the risk factors of hypertension severity.

## 2. Materials and Methods

### 2.1. Sample Size Calculation

To investigate the distribution characteristics of MTHFR gene polymorphism, especially the distribution frequency of the TT type gene in the Shanxi province of northern China, we conducted this cross-sectional survey. According to the similar findings in the neighboring provinces of Shanxi, the TT gene has a distribution frequency of about 30%. According to the admissible error of 3% and confidence of 1 − *α* = 0.95, PASS15 software was used to calculate the sample size. The result showed that the sample size was 928. Since the information of the research subjects was all obtained from the hospital medical record system and the data was high availability, it could be assumed that the data unavailability rate was 5%. Thus the sample size *N*=928/0.95=976 cases were required.

In the second part of this study, we aim to study polymorphisms of MTHFR C677T and the severity of hypertension, which is an exploratory study. The sample size should be no less than 15–20 times the number of independent variables. According to the research plan, the number of independent variables is 17; the sample size of this part of the study should range from 255 to 340 cases.

### 2.2. Study Population

In the present retrospective study, 985 unrelated subjects (617 males and 368 females) were recruited from individuals who visited the Second Hospital of Shanxi Medical University between January 2017 and January 2018, which could meet the research needs of sample size. Participants who met the following criteria were enrolled: (i) Age, ≥18 years; (ii) nonimmigrant and resident in Shanxi Province for at least 2 years; (iii) unrelated Han ethnic. The exclusion criteria were as follows: (i) those who had taken folic acid tablets, or vitamin B/multivitamins supplements within the preceding 2 weeks; (ii) complicated with a tumor, hepatic dysfunction, renal insufficiency, or thyroid dysfunction.

Additionally, 306 subjects with EH were selected from the 985 subjects according to the hospital medical records system to study polymorphisms of MTHFR C677T and the severity of hypertension, which could meet the needs of the sample size. These participants were required to meet the following criteria: selected according to the 2018 Chinese Guidelines for Prevention and Treatment of Hypertension, and a clinical SBP ≥140 mmHg and/or DBP ≥90 mmHg without the use of antihypertensive medications in three separate visits on different days. Patients with cardiac insufficiency, hyperuricemia, diabetes, and simple systolic hypertension were excluded.

The present study was approved by the Ethics and Research Committee of the Second Hospital of Shanxi Medical University for Experiments involving humans.

### 2.3. Sample Sources

The whole blood samples of the surveyed population were obtained from the biological sample bank at the Department of Pharmacy of the Second Hospital of Shanxi Medical University. The biological sample bank contains samples from people who have been undergoing blood concentration monitoring and genetic testing in the laboratories of the Department of Pharmacy since 2016.

### 2.4. Demographic Information and Biochemistry Index Collection

As part of the patient's hospital visits information, the basic demographic data of the participants (including gender, age, career, marital status, residence, smoking, alcohol consumption, family history, current medical history, past medical history, medication status, etc.) were collected from the hospital medical records system. In addition, the biochemistry index, including total cholesterol (TC), triglycerides (TG), low-density lipoprotein cholesterol (LDL-C), alanine aminotransferase (ALT), aspartate transaminase (AST), gamma-glutamyl transpeptidase (GGT), and homocysteine (Hcy), were collected from the hospital laboratory test system.

All staff has been specially trained in the early stage of the study, and the method of double inquiry and double input was adopted to ensure the authenticity and reliability of the data.

### 2.5. Genotyping of MTHFR Polymorphism

**Figure 1:**
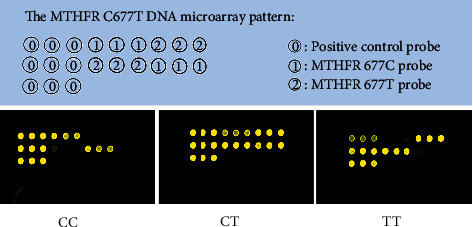
Microarray visualizations diagrams of the MTHFR C677T DNA.

DNA was extracted from whole blood samples using a DNA extraction kit, according to the instructions (BaiO Technology Co, Ltd.). Genotyping of MTHFR C677T polymorphisms was performed using PCR amplification and microarrays using commercially available kits (BaiO Technology Co., Ltd.). A 25-*μ*L PCR reaction mixture contained 20 ng DNA template and the recommended amounts of primers, dNTPs, and Taq DNA polymerase. The PCR thermocycling conditions were as follows: Predenaturing at 94°C for 5 minutes, followed by 35 cycles of 94°C for 25 seconds, 56°C for 25 seconds, and 72°C for 25 seconds, and a ﬁnal extension at 72°C for 5 minutes. The PCR products were then dispensed into a hybridization reaction chamber for hybridizing reactions. Based on the mutation from the wild type of MTHFR at position 677 from C to *T*, the MTHFR was divided into homozygous C allele (CC), heterozygous (CT), and homozygous *T* allele (TT) genotypes. Genotypes of MTHFR C677T were visualized by using the BaiO Array Doctor Version 2.0 software and BaiOBE-2.0 software according to the manufacturer's protocol (BaiO Technology Co, Ltd.). The microarray visualizations diagrams of the MTHFR C677T were shown in Figure 1.

### 2.6. Blood Pressure Classifications

According to the Diagnostic Criteria of Chinese Guidelines for the Prevention and Treatment of Hypertension (2018 edition), individuals with hypertension were divided into 3 grades according to the severity of blood pressure: Grade 1, SBP 140∼159 mmHg and/or DBP 90∼99 mmHg; Grade 2, SBP 160∼179 mmHg and/or DBP 100∼109 mmHg; Grade 3, SBP ≥180 mmHg and/or DBP ≥110 mmHg.

### 2.7. Statistical Analyses

Statistical analyses were performed using Statistical Package for the Social Sciences (SPSS) version 20.0 statistics software (SPSS). A Shapiro-Wilk test was used to determine the distribution of the data. Continuous data that was normally distributed are expressed as the mean ± standard deviation and nonnormally distributed data are presented as the median ± interquartile range. A Kruskal-Wallis H test was used to compare the overall differences in Hcy levels amongst groups with different genotypes, and a Dunn's test was used to compare the Hcy levels of the CT and TT groups with those of the CC group. Categorical data were expressed as *n* (%). A *χ*^2^ was used to assess genotype and allele frequency differences between groups.

A *χ*^2^ test was used to assess the differences between categorical variables, and a Kruskal–Wallis H test was used to assess the differences between continuous variables. Allele frequency was determined by direct counting, and the standard goodness-of-ﬁt test was used for testing of Hardy–Weinberg equilibrium (HWE) using a Pearson *χ*^2^ test. A univariate logistical regression model was used to filter candidate variables. Candidate variables with *P* < 0.05 on the logistical regression were included in the ordinal multivariate logistical regression model. Variables for inclusion were carefully chosen, given the number of events available, to ensure the parsimony of the final model. The ordinal multivariate logistical regression model was used to estimate the odds ratio (OR) and 95% conﬁdence interval (CI) for the association between hypertension and the variables. A 2-tailed *P* < 0.05 was considered to indicate a statistically signiﬁcant difference.

## 3. Results

### 3.1. Distribution Genotypes and Allele Frequency of MTHFR C677T

A total of 985 Chinese Han (617 males and 368 females) from north China's Shanxi Province were included in the study. The average age of the subjects was 50.22 ± 19.93 (21 years to 90 years). The C677T genotype and allele frequency revealed no signiﬁcant departures from Hardy–Weinberg equilibrium in the population from the Shanxi region (*χ*^2^ = 0.125, *P* > 0.05) (Table 1).

Genotype and allele frequency of C677T polymorphism of MTHFR according to gender and age are shown in Table 2. In total, 492 (49.95%) subjects were heterozygous (CT); 300 (30.46%) subjects were homozygous *T* allele (TT), and 193 (19.59%) subjects were homozygous C allele (CC). The allele frequency of *T* mutation was 55.43%. According to the results, the total frequency of the MTHFR C677T genotype did not significantly differ between gender and age.

### 3.2. MTHFR C677T genetic polymorphism and Homocysteine (Hcy)

**Figure 2:**
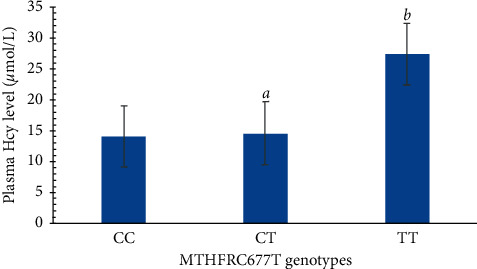
Plasma Hcy levels in patients with different genotypes of C677T. HCY levels were significantly different among the three groups (*P* < 0.001). Compared with CC genotype, ^a^*P* > 0.05; ^b^*P* < 0.05.

High levels of Hcy are a risk factor for cardiovascular and cerebrovascular diseases. In the present study, the median concentration of Hcy was 13.50 ± 7.95 *μ*mol/L (5.00 *μ*mol/L–86.40 *μ*mol/L). The proportion of individuals with an Hcy concentration of ≥10 *μ*mol/L accounted for 84.13%. The normality test showed that plasma Hcy levels in individuals with varying genotypes were not normally distributed. According to the Kruskal–Wallis H test, the overall difference in the Hcy level between different genotypes was statistically significant. A Dunn's test was used to compare the Hcy levels of CT and TT groups with those of CC groups, and the results showed that the plasma Hcy level of individuals with a TT genotype was higher than that of individuals with a CT and CC genotype, as shown in Figure 2. Hcy levels of different C677T genotypes were significantly different.

The study population was further divided *t* into three groups according to the plasma Hcy levels: the normal group (Hcy 5–10 *μ*mol/L); middle group (Hcy 10–15 *μ*mol/L), and the high Hcy group (Hcy ≥15 *μ*mol/L). As shown in Table 3, the distribution of individuals with a CC or CT genotype was relatively high in the middle group; individuals with a TT genotype were more prevalent in the high Hcy group, and the distribution frequency increased with Hcy (*χ*^2^_trend_ = 64.77, (*P* < 0.001)).

### 3.3. MTHFRC677T Genetic Polymorphism and Hypertension

A total of 306 patients with essential hypertension were enrolled in the study. The mean age was 65.50 ± 12.46 years. The number of patients with grade 1, 2, or 3 hypertension was 53 (17.32%), 90 (29.41%), and 163 (53.27%), respectively. The general baseline clinical characteristics of the patients are presented in Table 4. There were no significant differences in the baseline characteristics of participants with different hypertension grade, except for a family history of hypertension and smoking.

**Figure 3:**
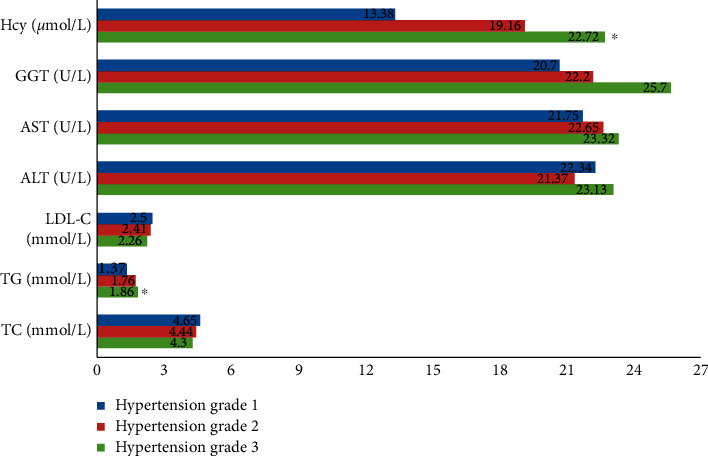
Levels of various biochemical indexes in different grades of hypertension. TG and HCY levels were significantly different in the three grades of hypertension. ^*∗*^*P* < 0.05. Hcy: homocysteine; TG: triglyceride; ALT: alanine aminotransferase; AST: aspartate transaminase; GGT: *γ*-glutamyl transpeptidase; LDL-C: low-density lipoprotein cholesterol; TC: total cholesterol.

**Figure 4:**
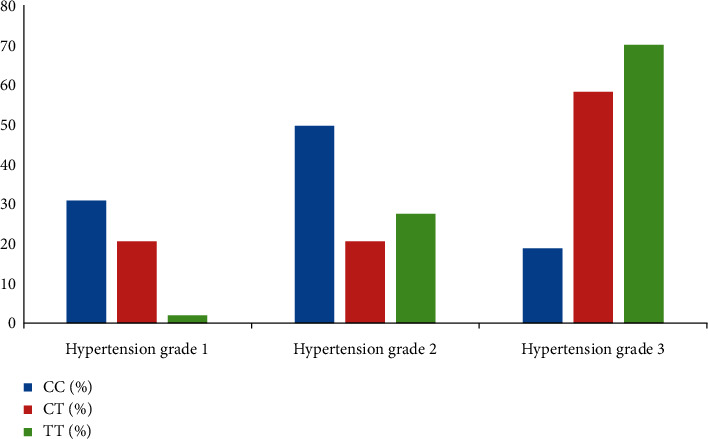
Distribution of the MTHFR C677T genotype in patients with different grades of hypertension. The TT genotype was widely distributed in patients with grade 3 hypertension, accounting for 70.2% of the individuals with a TT genotype. The CC genotype was more widely distributed in patients with grade 1 and 2 hypertension (30.9% and 50%, respectively).

As shown in Table 5, by comparing the biochemical indicators and distribution of the MTHFRC677T gene in patients with different grades of hypertension, excluding TG and HCY, the other biochemical indicators, including TC, LDL, ALT, AST, and GGT, were not associated with the severity of hypertension. The levels of various biochemical indicators and the distribution of MTHFR C677T genotype in different grades of hypertension are shown in Figures 3 and 4.

Ordered multiple classification logistic regressions analysis was used to identify risk factors associated with the severity of hypertension. The significant factors influencing the severity of hypertension in the single factor analysis were included in the ordered multiple classification logistic regression analysis. As shown in Table 6, smoking, family history of hypertension, MTHFRC677T genotype, Hcy levels, and TG were independent risk factors associated with the severity of hypertension. Compared with nonsmokers and individuals without a family history of hypertension, smoking, and family history of hypertension increased the risk of a one-level rise in the severity of hypertension by 2.04 and 1.81 times. Compared with MTHFR677CC, MTHFR677CT and MTHFR677TT also increased the risk by 2.29 and 2.24 times, respectively. Furthermore, the results also showed that Hcy and TG could also increase the risk of hypertension (OR = 1.04 and 1.26, respectively).

## 4. Discussion

Methylene-tetrahydrofolate reductase, a key enzyme involved in Hcy metabolism, is associated with the occurrence and development of several diseases. Identiﬁcation of a genetic predisposition to these diseases may be beneficial for the development of personalized risk forecasting and health care strategic planning. In the present study, the genetic polymorphisms of MTHFR C677T and its relationship with the severity of hypertension in a cohort recruited from the Shanxi region were assessed. The reported data could be useful in guiding health decisions to delay or halt EH in susceptible individuals.

The polymorphisms of MTHFR C677T was analyzed in 985 individuals from the Shanxi Province. The results indicated that 19.59% of individuals were wild-type homozygous (CC), 49.95% were heterozygous (CT), and the remaining individuals (30.46%) were mutant allele homozygous (TT). Previous studies on MTHFR gene polymorphism have shown considerable heterogeneity between different countries, and similar results have been observed in the Chinese population [2, 3]. This present study showed that in north China the distribution of TT genotype in Shanxi was similar to that in Tianjin (30.4%) and Shaanxi (30.4%), lower than that in Shandong (40.8%) and Henan (37.0%), and higher than that in some of China's southern provinces such as Jiangsu (19.7%), Hubei (16.8%), Sichuan (13.8%), Yunnan (15.3%), Guangdong (8.3%), and Hainan (6.4%) [12]. Furthermore, whilst the majority of studies reported no differences in MTHFR C677 T gene polymorphisms by sex [12, 13], other studies have shown that the distribution frequency of CT and TT was higher in Chinese women compared with men [14]. In the present study, there were no differences in the distribution of MTHFR genotypes based on sex or age, consistent with the majority of most previous studies.

The increase of the Hcy level caused by the mutation of the MTHFR C677T gene is one of the most frequent genetic factors that can increase the risk of disease. The results of the present study suggested that the percentage of the population with Hcy concentration ≥10 *μ*mol/L was as high as 84.13%. Furthermore, the correlation analysis of MTHFRC677T polymorphism and Hcy showed that the average Hcy levels in patients with the homozygous TT genotype were significantly higher than that of patients with CT or CC genotype. Further subgroup analyses were performed based on the Hcy levels, and the results showed that the TT genotype had the highest distribution frequency (63.7%) in the high Hcy group. The results suggested that MTHFRC677T polymorphism may be an essential factor for determining the Hcy levels in Chinese individuals, consistent with most relevant studies [15, 16].

Previous genetic epidemiologic studies have shown that the genetic variants in the MTHFR gene may be associated with a variety of diseases, including ischemic stroke [17, 18], coronary heart disease [19], hypertension [20, 21], amongst other diseases; however, their mechanisms remain unclear. Based on the present study, the common C677T mutant of the MTHFR gene is located in the catalyzed region and can cause an increase in blood Hcy concentrations. Elevated plasma Hcy levels increase oxidative stress and result in damage to the vascular endothelium, ultimately leading to an imbalance of antioxidant status and endothelial dysfunction [22, 23]. Endothelial dysfunction and imbalance in antioxidant status have been associated with the pathogenesis of hypertension [24]. Reducing Hcy levels improve BP, suggesting a close relationship between Hcy and BP levels [25]. In the present study, Hcy concentrations and the distribution of the MTHFR C677T gene polymorphisms were significantly different between patients with different hypertension grades. Patients with grade 3 hypertension were more likely to have MTHFR C677T genotypes of CT or TT and higher Hcy concentrations. Conversely, the distribution frequency of individuals with a CT or TT genotype and the prevalence of individuals with a high concentration of Hcy were relatively lower in the grade 1 hypertensive group.

Further analysis showed that Hcy, MTHFR677CT, and MTHFR677TT were associated with an increased risk and severity of hypertension (OR = 1.04, 2.29, 2.24). In combination with our previous studies, we found that C677T mutations common in the MTHFR gene lead to elevated plasma Hcy concentrations. Hcy can be converted into an effective cell methylation inhibitor S-adenosylhomocysteine (SAH) [26]. Increased SAH concentrations may result in a decrease of s-adenosine methionine (Sam) and hypomethylation of genomic DNA. In turn, genomic DNA hypomethylation can promote the expression of certain genes, thereby activating the renin-angiotensin-aldosterone system and leading to hypertension [27]. This might be another mechanism through which the MTHFR C677T gene mutation increases the severity of hypertension.

The severity of hypertension was positively associated with smoking, family history of hypertension, and TG levels. Hcy levels in smokers with hypertension are higher compared with nonsmokers with hypertension [28]. Thus, it was hypothesized that smoking may increase Hcy levels in hypertensive patients and affect the severity of hypertension. It is well known that MTHFR C677T polymorphisms are associated with increased Hcy concentrations and lower folate levels [29, 30]. Individuals who are deficient in folate are at a higher risk of developing hypertriglyceridemia [31]. Furthermore, Huang et al. found that individuals with the MTHFR 677CT/TT genotype have higher levels of triglycerides and total Hcy levels compared with individuals with a wild-type genotype in patients with hyperlipidemia from northern China [32]. In the present study, the MTHFR 677CT/TT genotypes were determined to be a risk factor for the severity of hypertension. It was hypothesized that the mutations of MTHFR C677T, which resulted in the elevated TG levels, may aggravate the degree of hypertension. In addition, it has been shown that a family history of hypertension is an independent risk factor for the severity of hypertension, and the incidence of grade 3 hypertension is higher in individuals with a family history of hypertension [33], consistent with the results of the present study.

The present study has a few limitations. First, the participants' plasma folate levels were not measured, so it was not possible to determine the association between MTHFR C677T polymorphism and folate levels, and whether the polymorphisms and folic acid levels affected the outcomes of this study. Second, all the enrolled individuals were from a tertiary hospital in Shanxi Province. The possibility that individuals may choose hospitals according to their geographical location was not considered; therefore, a potential selection bias may exist. Third, the present study focused on the outcomes related to the severity of hypertension. Whether the polymorphisms had additional effects on SBP or DBP was not considered. Finally, although the present study conﬁrmed that higher circulating Hcy levels were associated with the severity of hypertension, and although there are several studies suggesting that Hcy-lowering is beneficial in the treatment of hypertension, the efﬁcacy of Hcy-lowering therapy for the prevention and treatment of hypertension remains to be evaluated.

The present study revealed a high MTHFR 677TT gene distribution of individuals from the Shanxi province of China. Homozygous TT genotype carriers of the MTHFR gene had signiﬁcantly higher plasma Hcy levels and were at an increased risk of hypertension. In addition, according to the results, smoking, family history of hypertension, and higher TG levels were considered risk factors for the development of severe hypertension. Identification of populations at high risk of hypertension may facilitate the development of individualized strategies for the management of hypertension in the studied population.

## 5. Conclusions

The present study assessed the polymorphic distribution of MTHFR C677T in a large population living in northern China. High-risk factors of hypertension in Chinese individuals were identified that can significantly promote the development of hypertension control strategies.

## Figures and Tables

**Table 1:** Hardy–Weinberg equilibrium test for the methylenetetrahydrofolate reductase polymorphisms.

MTHFR C677T	Actual frequency (%)	Theoretical frequency (%)	*χ* ^2^	*P* value
CC	193 (19.59)	196.0 (19.89)	0.125	0.944
CT	492 (49.95)	486.6 (49.42)		
TT	300 (30.46)	302.4 (30.69)		
Total	985	985		

**Table 2:** Genotypes and allele frequency of methylenetetrahydrofolate reductase C677T in theG cohort recruited.

Group	*N*	Genotype frequency (%)			Allele frequency (%)		*P* value
		CC	CT	TT	C	T	
Total	985	193	492	300	878	1092	
		(19.59)	(49.95)	(30.46)	(44.57)	(55.43)	
Gender							0.053
Males	617	130	290	197	550	684	
		(21.07)	(47.00)	(31.93)	(44.57)	(55.43)	
Females	368	63	202	103	328	408	
		(17.12)	(54.89)	(27.99)	(44.57)	(55.43)	
Age							0.363
<45	143	25	76	42	126	160	
		(17.48)	(53.15)	(29.37)	(44.06)	(55.94)	
45–59	390	77	205	108	359	421	
		(19.74)	(52.56)	(27.69)	(46.03)	(53.97)	
≥60	452	91	211	150	393	511	
		(20.13)	(46.68)	(33.19)	(43.47)	(56.53)	

CC is the “normal” genotype, CT the heterozygote, and TT the homozygote abnormal.

**Table 3:** Distribution of methylenetetrahydrofolate reductase 677T genotypes based on Hcy levels.

Hcy (*µ*mol/L)	*N*	Genotype frequency (*n*) (%)^a^			*χ* ^2^	*P* value
		CC	CT	TT		
5–10	179	46 (25.70)	106 (59.22)	27 (15.08)	99.81	<0.001
10–15	409	90 (22.00)	237 (57.95)	82 (20.05)		
≥15	397	57 (14.36)	149 (37.53)	191 (48.11)		

^a^ *χ* ^2^_trend_ = 64.77, (*P* < 0.001). Hcy: homocysteine.

**Table 4:** Univariate analysis of characteristics associated with essential hypertension.

Characteristic	Hypertension grade			*P* value
	Grade 1	Grade 2	Grade 3	
Sex *n* (%)				0.508
Male	34 (16.3)	59 (28.2)	116 (55.5)	
Female	19 (19.6)	31 (32.0)	47 (48.5)	
Age, years^b^	65.28 ± 15.02	67.86 ± 10.41	64.28 ± 12.47	0.609
BMI (kg/m^2b^)	25.22 ± 4.24	24.87 ± 3.12	25.25 ± 3.08	0.584
Family history of hypertension *n* (%)				0.009^a^
No	43 (21.4)	62 (30.8)	96 (47.8)	
Yes	10 (9.5)	28 (26.7)	67 (63.8)	
Career *n* (%)				0.544
Unemployed	5 (13.5)	12 (32.4)	20 (54.1)	
Retired	29 (16.5)	56 (31.8)	91 (51.7)	
Manager	8 (20.5)	8 (20.5)	23 (59.0)	
Technician	3 (16.7)	2 (11.1)	13 (72.2)	
Other	8 (22.2)	12 (33.3)	16 (44.4)	
Marital status *n* (%)				0.145
Married	49 (18.6)	80 (30.3)	135 (51.1)	
Divorced/widowed	4 (9.5)	10 (23.8)	28 (66.7)	
Residence *n* (%)				0.978
City	42 (17.5)	70 (29.2)	128 (53.3)	
Countryside	11 (16.7)	20 (30.3)	35 (53.0)	
Smoking *n* (%)				0.001^a^
Yes	13 (9.2)	41 (29.1)	87 (61.7)	
No	40 (24.2)	49 (29.7)	76 (46.1)	
Drinking *n* (%)				0.976
Yes	18 (17.5)	31 (30.1)	109 (52.4)	
No	35 (17.2)	59 (29.1)	54 (53.7)	

^a^ *P* < 0.01. ^b^Mean ± SD. BMI body mass index.

**Table 5:** Univariate analysis of biochemical indicators and MTHFR C677T polymorphisms in patients with hypertension.

Factor	Hypertension grade (%)			*P* value
	Grade 1	Grade 2	Grade 3	
TC (mmol/l)	4.65 ± 1.20	4.44 ± 0.96	4.30 ± 1.01	0.131
TG (mmol/l)	1.37 ± 0.48	1.76 ± 0.81	1.86 ± 1.05	0.028^a^
LDL-C (mmol/l)	2.50 ± 0.78	2.41 ± 0.66	2.26 ± 0.81	0.050
ALT (U/l)	22.34 ± 16.35	21.37 ± 11.45	23.13 ± 15.40	0.702
AST (U/l)	21.75 ± 10.40	22.65 ± 6.85	23.32 ± 8.10	0.725
GGT (U/l)	33.21 ± 17.85	30.74 ± 17.38	35.60 ± 19.70	0.069
Hcy (*μ*mol/l)	13.38 ± 5.60	19.16 ± 9.72	22.72 ± 14.30	0.000^c^
MTHFR C677T genotype (*n*) (%)				
CC	21 (30.9)	34 (50.0)	13 (19.1)	0.005^b^
CT	30 (20.8)	30 (20.8)	84 (58.4)	
TT	2 (2.1)	26 (27.7)	66 (70.2)	

^a^ *P* < 0.05^b^*P* < 0.01^c^*P* < 0.001 MTHFR: methylenetetrahydrofolate reductase; TG: triglycerides; TC: total cholesterol; LDL-C: low-density lipoprotein cholesterol; ALT: alanine aminotransferase; AST: aspartate transaminase; GGT: *γ*-glutamyl transpeptidase.

**Table 6:** Multivariate logistical regression analysis for hypertension classification.

Variables	*β*	OR (95% CI)	*P* value
Smoking			
Yes	0.711	2.04 (1.29–3.22)	0.002^b^
No	—	1	—
Family history of hypertension			
Yes	0.595	1.81 (1.11–2.97)	0.018^a^
No	—	1	—
MTHFR C677T genotype			
TT	0.807	2.24 (1.14–4.39)	0.019^a^
CT	0.83	2.29 (1.29–4.08)	0.005^b^
CC	—	1	—
Homocysteine (*μ*mol/l)	0.035	1.04 (1.01–1.06)	0.003^b^
Triglycerides (mmol/l)	0.23	1.26 (1.00–1.58)	0.047^a^

^a^ *P* < 0.05. ^b^*P* < 0.01. OR: odds ratio; CI: confidence interval; MTHFR: methylenetetrahydrofolate reductase.

## Data Availability

The datasets used and/or analyzed during the current study are available from the corresponding author on reasonable request.
